# Optimization technique combined with deep learning method for teeth recognition in dental panoramic radiographs

**DOI:** 10.1038/s41598-020-75887-9

**Published:** 2020-11-06

**Authors:** Fahad Parvez Mahdi, Kota Motoki, Syoji Kobashi

**Affiliations:** Graduate School of Engineering, University of Hyogo, 2167 Shosha, Himeji, Hyogo 671-2280 Japan

**Keywords:** Engineering, Mathematics and computing

## Abstract

Computer-assisted analysis of dental radiograph in dentistry is getting increasing attention from the researchers in recent years. This is mainly because it can successfully reduce human-made error due to stress, fatigue or lack of experience. Furthermore, it reduces diagnosis time and thus, improves overall efficiency and accuracy of dental care system. An automatic teeth recognition model is proposed here using residual network-based faster R-CNN technique. The detection result obtained from faster R-CNN is further refined by using a candidate optimization technique that evaluates both positional relationship and confidence score of the candidates. It achieves 0.974 and 0.981 mAPs for ResNet-50 and ResNet-101, respectively with faster R-CNN technique. The optimization technique further improves the results i.e. *F*_*1*_ score improves from 0.978 to 0.982 for ResNet-101. These results verify the proposed method’s ability to recognize teeth with high degree of accuracy. To test the feasibility and robustness of the model, a tenfold cross validation (CV) is presented in this paper. The result of tenfold CV effectively verifies the robustness of the model as the average *F*_*1*_ score obtained is more than 0.970. Thus, the proposed model can be used as a useful and reliable tool to assist dental care professionals in dentistry.

## Introduction

Visual examination by dental care experts during dental treatment alone cannot provide sufficient information to diagnose a number of dental anomalies. It is because of their location in the mineralized tissues (bone and teeth). Thus, it is indispensable to use digital radiographs during dental treatment. Immediate availability of digital images, limited radiation dose and the possibility of applying image processing techniques (such as image enhancement and image registration) are some of the advantages of dental radiographs. However, the possibility of computer aided analysis of digital radiographs is one of the most important aspects of using dental radiographs. Dental panoramic radiograph or simply pantomograph is a type of dental radiographs that provides the dentist an unobstructed view of the whole dentition (both upper and lower jaws). It contains detail information of the dentomaxillofacial anatomy^1^.

The usage of dental radiographs is growing day by day and therefore, it is highly desirable to assist dentist with computer-aided analysis. Automatic recognition of teeth from dental radiographs can be a great way to aid dentists in dental treatment. It will not only reduce workload from dental professionals but also reduce interpretation error and diagnosis time, and eventually will increase the efficiency of dental treatment. Usage of machine learning (ML) and computer vision techniques is not new in dental radiographs. Nomir et al.^2^ proposed an automatic system that can identify people from dental radiographs. The proposed system can segment the radiographs into individual teeth automatically, represent and match teeth contours and finally provides matching scores between antemortem (AM) and postmortem (PM) teeth. Nassar et al.^3^ created a prototype architecture of automated dental identification system (ADIS) to address the problem of postmortem identification through matching image feature. This matching problem was tackled by high level feature extraction in the primary step to expedite retrieval of potential matches followed by image comparison using inherent features of dental images. To detect areas of lesions in dental radiographs, a semiautomatic framework is proposed by Li et al.^4^ by using level set method. The framework, at first, segments the radiograph into three meaningful regions. It was done by using two coupled level set functions. Then, an analysis scheme influenced by a color emphasis scheme prioritizes radiolucent areas automatically. After that the scheme employed average intensity profile based method to isolate and locate lesions in teeth. The framework improved the interpretation in a clinical settings and enables dentist to focus their attention on critical areas. Local singularity analysis based teeth segmentation was proposed by Lin et al.^5^ in periapical radiographs in order to detect periapical lesion or periodontitis. This method works on four different stages that include adaptive power law transformation (as image enhancement technique), Hölder exponent (for local singularity analysis), Otsu’s thresholding and connected component analysis (as tooth recognition) and finally, snake boundary and morphological operations (for tooth delineation). The overall accuracy (considering true positive) was found to be near 90 percent.

Kavitha et al.^6^ employed a new support vector machine (SVM) method to diagnose osteoporosis (a disease that increases the risk of fractures in bone) at early stage to reduce the risk of fractures. They utilized dental panoramic radiographs to measure the thin inferior cortices of mandible which is very useful to identify osteoporosis in women.

The limitation of the conventional ML techniques in processing raw natural data requires careful engineering to construct feature extractor in order to transform the raw data into a suitable representation for detecting or classifying input patterns. This limitation was overcome effectively by the introduction of deep learning techniques. Deep learning techniques are representation learning based techniques that allow a machine to be fed with raw data and then process the data in different layers to automatically discover necessary representations to detect or classify input data. The main advantage of deep learning is that these layers of features are learned directly from the raw data by using a general purpose learning procedure instead of design constructed by the human engineers^7^. It has thus caused remarkable improvements in artificial intelligence. Deep learning techniques beat records in image^8^ and speech recognitions^9^, supersedes other machine learning techniques in analyzing particle accelerator data^10^, predicting activity in potential drug molecules^11^, reconstructing brain circuits^12^, and produced promising results in natural language understanding^13^.

Transfer learning based convolutional neural network (CNN) was utilized by Prajapati et al.^14^ to classify three kinds of dental diseases from dental radiographs. They have utilized a pretrained VGG16^15^ as feature detector. Lin et al.^16^ proposed an algorithm based on CNN to automatically detect teeth and classify their conditions in panoramic dental radiographs. In order to increase the amount of data, different data augmentation techniques such as flipping and random cropping are used. They claimed to achieve accuracy around 90% using different image enhancement techniques along with CNN. Chen et al.^17^ proposed faster R-CNN technique that included three post processing steps on dental periapical films. The post processing steps included a filtering system, a neural network model and a rule-base module to refine and supplement faster R-CNN. Although, the detection rate was exceptionally well, there classification result was only very close to the level of a junior scientist even after applying three post-processing steps. Tuzoff et al.^18^ proposed a model that used faster R-CNN for teeth detection, VGG-16 based convolutional network for classification and heuristic-based algorithm for result refinement. Although, their heuristic algorithm heavily depended on the confidence scores produced by the convolution network, adequate performance analysis of the convolutional network for teeth classification was not present. Muramatsu et al.^19^ proposed a fully convolutional network (FCN) based on GoogleNet to detect teeth. A ResNet-50 based pretrained network was then used to classify tooth by its type i.e. incisors, canines, premolars, and molars as well as three different tooth conditions. They tried to improve the classification result by introducing double input layers with multisized image data. However, their final classification result i.e. 93.2% for teeth classification was fallen short to the required accuracy needed for clinical implementation.

This paper proposes a residual network based faster R-CNN algorithm in panoramic radiographs for automatic teeth recognition. Faster R-CNN object detector is the modified and upgraded version of R-CNN^20^ and fast R-CNN^21^. The main advantage of faster R-CNN is that it does not need a separate algorithm for region proposals; rather the same convolution network is used for region proposal generation and object detection and hence much faster than its predecessors are. Two variants of trained residual network i.e. ResNet-50 and ResNet-101 are utilized in this paper to increase the effectiveness of the proposed system. Residual network is widely known for mitigating infamous vanishing gradients problem in deep network^22^. This paper proposes a candidate optimization algorithm based on prior knowledge of the dataset to further refine the detection results obtained by residual network based faster R-CNN. The proposed candidate optimization method considers both the position patterns of detected boxes as well as the confidence scores of the candidates given by the faster R-CNN algorithm to refine the detected boxes. The proposed method, thus, combines an optimization algorithm with deep learning technique for teeth recognition in dental panoramic radiographs.

The rest of the paper is structured as follows; “Materials” section describes about the data i.e. dental radiographs used in this research. It also describes the tooth numbering systems used for testing the performance of the proposed method. “Proposed method” section presents the method and the architecture of the proposed model as well as explanation of training and test datasets. It also includes description of the candidate optimization method proposed in this paper. “Results and discussions” section comprises of results and discussions include simulation results. Finally, the paper is concluded with the “conclusion” section, providing the gist of the paper and possible future work.

## Materials

**Figure 1:**
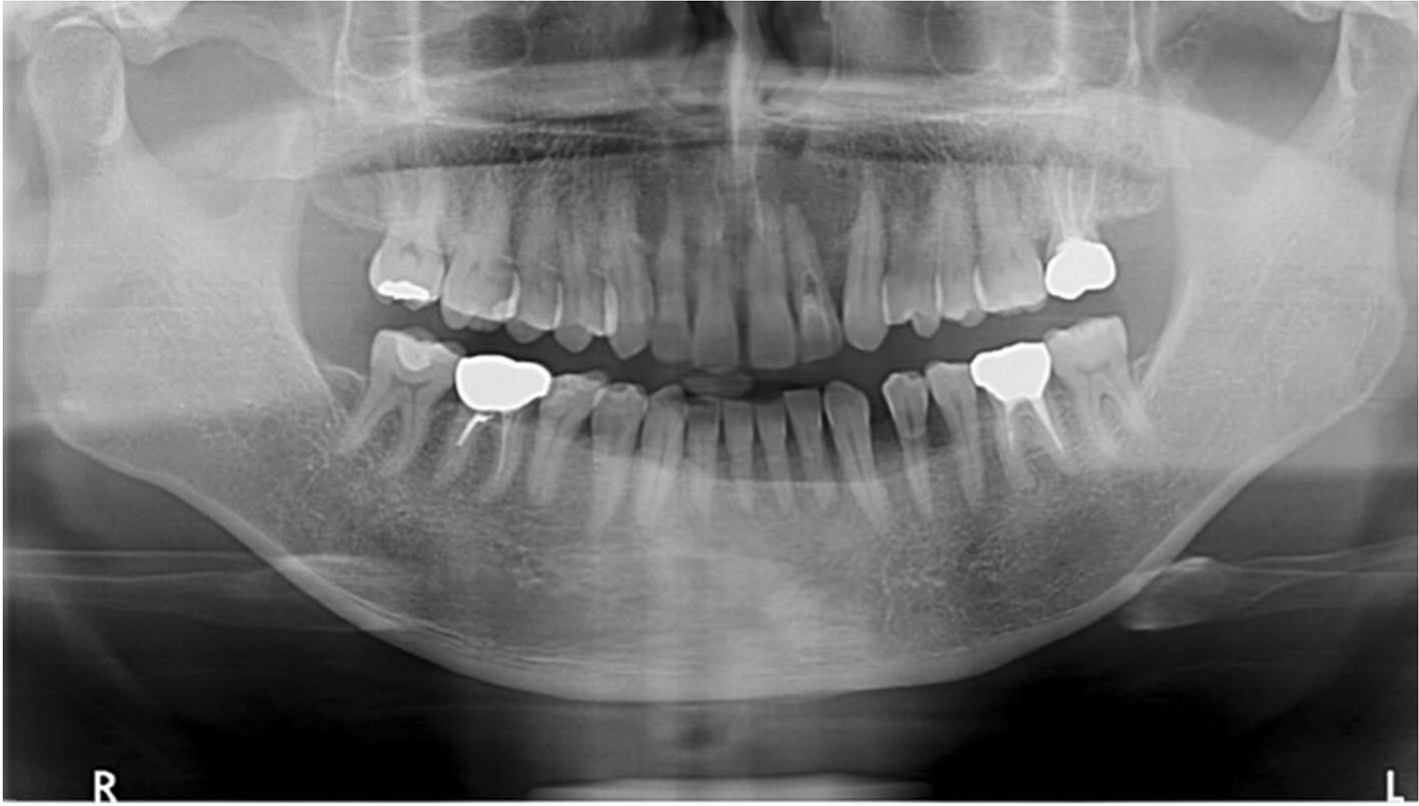
Dental panoramic radiograph or pantomograph.

**Figure 2:**
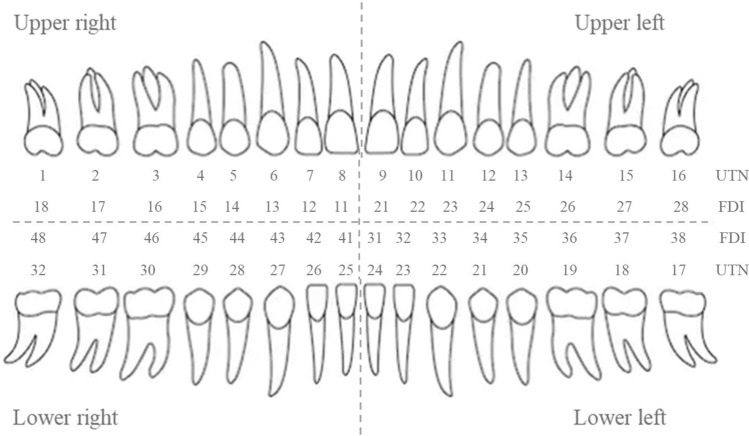
Universal tooth numbering (UTN) system and Fédération Dentaire Internationale (FDI) system.

A total of 1000 panoramic radiographs were collected for this research. The dimension of the images was around (1400–3100) × (800–1536) pixels and stored as a jpeg format. The images were collected by Narcohm Co. Ltd. from multiple dental clinics under the approval of each clinic. The authors obtained the images from Narcohm Co. Ltd. with permission. Also, the images were anonymously collected so that no additional information, like age, gender or height was revealed. Figure 1 shows an example of a collected dental panoramic radiograph. For the sake of training and validation, all the images were labeled by putting a rectangular bounding box around each tooth with proper roots and shape. The panoramic radiographs consisted of normal teeth, missing teeth, residual roots and dental implants. This paper followed universal tooth numbering (UTN) system. In universal tooth numbering system, teeth count starts from upper right part to upper left part as 1 to 16 and then lower left to lower right as 17 to 32. Fédération Dentaire Internationale (FDI)^23^ and Palmer notation (PN)^24^ are other two notable tooth numbering systems. Figure 2 illustrates the UTN and FDI system simultaneously.

Annotated 1000 radiographs were split into total tenfolds, each containing 100 radiographs; (1) training dataset consisted of total ninefolds and (2) test dataset consisted of onefold. Therefore, training and test datasets were consisted of 900 radiographs and 100 radiographs, respectively. Training dataset was used to train faster R-CNN, whereas test dataset was used to analyze and validate the performance of the proposed method.

## Proposed method

The proposed method consists of two steps; (i) candidate detection and (ii) optimization. The first step detects candidates from the panoramic radiographs using faster R-CNN and in the second step, the detected candidates are refined using an optimization method. Image-based CNN detectors are used in this research and therefore, a brief overview of these detectors are given at first. Next, deep learning-based faster R-CNN technique, its architecture and criteria of introducing transfer learning techniques are presented and discussed. Finally, the proposed optimization method based on prior knowledge is explained along with brief descriptions of performance evaluation metrics.

### Image-based CNN detectors and candidate detection using faster R-CNN

Mainly two types of image-based CNN detectors have been developed, they are (i) single-stage methods and (ii) two-stage methods. Both types of method utilize multiple feature maps of different resolutions for object detection. These feature maps are generated by a backbone network e.g. AlexNet^8^ or ResNet^22^.

The single-stage name came from the fact that these kind of methods performed directly into these multi-scale feature maps for object detection. In contrast, two-stage methods at first work on the feature maps to generate region proposals from the anchor boxes. Anchor boxes are a set of predefined bounding boxes with different aspect ratio. The network that generates region proposals is known as region proposal network (RPN) in faster R-CNN. RPN produces region proposals by predicting whether the anchor boxes contain an object or not (without classifying which object). Region proposals with best confidence scores are then processed into the second stage for further classification and regression. Thus, the region proposals are classified and regressed twice and that is why usually these kinds of methods achieve higher accuracy. However, the second stage computation adds an extra computational burden and the system, thus, tends to be less efficient and slow^25^.

In this research, faster R-CNN^26^ technique is used for automatic teeth recognition. Faster R-CNN is modified and updated version of fast R-CNN^21^. It utilizes two different modules; one is deep convolutional network also known as region proposal network (RPN) for region proposals and second is fast R-CNN object detector that utilizes proposed regions. Two modules, however, work as single unified network for object detection. The main advantage of faster R-CNN from its predecessors R-CNN^20^ and fast R-CNN is that it successfully alleviate the problem of needing a separate algorithm for region proposals and thus enabling a cost effective region proposals. A single unified network is used for both region proposals and object detection. The technique won 1st place in several tracks e.g. ImageNet detection, ImageNet localization, COCO detection and COCO segmentation of ILSVRC and COCO competitions^26^.

#### Transfer learning

Transfer learning is a technique used in both machine learning and deep learning problems to improve the learning performance of a particular task through transferring the knowledge gained from a different task that has already been learned. Usually it works best when the tasks are quite similar. However, it has been found that this technique works well even though the tasks are completely different. There are many pre-trained architectures that are trained on huge datasets such as AlexNet^8^, VGG-16^15^, VGG-19^15^, Inception-V3^27^, ResNet-50^22^ and ResNet-101^22^. Probably the most popular such dataset is ImageNet. It contains millions of data sample to classify 1000 different categories. Transfer learning technique enables the researchers to train models with minimal training data by fetching architecture and weights from some popular pre-trained model. Furthermore, it drastically reduces the computational cost and therefore the training time.

#### ResNet-50 & ResNet-101

In order to construct the proposed model, the ResNet-50 and the ResNet-101 architectures are adopted by the faster R-CNN framework separately, i.e. the proposed model utilizes both the architectures separately for teeth recognition task. He et al.^22^ presented a framework based on residual learning to overcome the difficulty of training deeper neural network. The degradation problem was addressed by introducing a deep residual learning framework. It showed that optimizing residual mapping is easier than to optimize the original and therefore, gained accuracy easily from network with greater depth. Residual network won the 1st place in classification task of ILSVRC 2015 competition^22^. ResNet-50 consists of four stages with total 50 layers and hence its name. ResNet-101 is the deeper version of ResNet-50, consisting of additional 17 blocks (3-layer block) in the third stage that made it total 101 layers. The architecture of ResNet-50 and ResNet-101 is summarized in Table 1.

**Table 1:** Architectures of ResNet-50 and ResNet-101.

Layer name	Output size	ResNet-50	ResNet-101
conv1	112 × 112	7 × 7, 64	
conv2_x	56 × 56	3 × 3 max pool	
		$$\left[\begin{array}{c}1\times 1, 64\\ 3\times 3, 64\\ 1\times 1, 256\end{array}\right]$$×3	$$\left[\begin{array}{c}1\times 1, 64\\ 3\times 3, 64\\ 1\times 1, 256\end{array}\right]$$×3
conv3_x	28 × 28	$$\left[\begin{array}{c}1\times \mathrm{1,128}\\ 3\times \mathrm{3,128}\\ 1\times 1, 512\end{array}\right]$$×4	$$\left[\begin{array}{c}1\times 1, 64\\ 3\times 3, 64\\ 1\times 1, 256\end{array}\right]$$×4
conv4_x	14 × 14	$$\left[\begin{array}{c}1\times \mathrm{1,256}\\ 3\times 3, 256\\ 1\times 1, 1024\end{array}\right]$$×6	$$\left[\begin{array}{c}1\times \mathrm{1,256}\\ 3\times 3, 256\\ 1\times 1, 1024\end{array}\right]$$×23
conv4_x	7 × 7	$$\left[\begin{array}{c}1\times \mathrm{1,512}\\ 3\times 3, 512\\ 1\times 1, 2048\end{array}\right]$$×3	$$\left[\begin{array}{c}1\times \mathrm{1,512}\\ 3\times 3, 512\\ 1\times 1, 2048\end{array}\right]$$×3
avg_pool, fc1000, fc1000_softmax	1 × 1	Average pool, classification, softmax	

#### Activation layer selection

In order to use a pre-trained model for a completely different task, few pre-processing steps should be considered. The steps include the removal of the original classifier, add a new classifier according to the task and fine tune the model^28^. There are three strategies to fine tune the model.

(i) The first strategy is to train the entire model, i.e. use only the architecture of the pre-trained model and train the model according to the available dataset. In short, training from the scratch. In order to achieve sufficient accuracy, large dataset is required for this strategy. It also involves huge computational cost.
(ii) The second option is to train some layers of the model, while leaving other frozen. In general, lower layers keep information about general features, whereas the higher layers keep information about specific features. As general features are problem independent, lower layers can be left frozen in case of small dataset. The training then only be done in the higher layers (problem dependent). However, when large dataset is available, overfitting does not become an issue and lower layers can also be train with the higher layers.
(iii) Third option is to freeze all the convolutional layers and thus, use only the classifier. This option can should only be considered where dataset is small and sufficient computational power is unavailable.

Based on the above strategies, activation layer 40 (activation_40_relu) is used as a feature extraction layer for ResNet-50 and activation layer res4b22 (res4b22_relu) for ResNet-101.

### Candidate optimization

The residual network based faster R-CNN together with careful selection of parameters can provide very good recognition performance. However, there may still be a good number of false positives including double detections for a single tooth. In order to cope up with this problem, a candidate optimization algorithm based on prior knowledge is proposed in this research. This model selects the best combination of candidates in order to filter out the false positives and thus improving overall efficiency of the model.

Assume that tooth *x* ($1\le x\le 32$) has $N\left(x\right)$ candidates detected by faster R-CNN. And, in some case, all candidates are false positives. Therefore, the selection is to find the best combination of candidates in ${\prod }_{x=1}^{32}\left(N\left(x\right)+1\right)$ combinations. The selection is done by optimizing Eq. (1). In this equation, the first term evaluates the confidence score, and the second term evaluates the relational position from other teeth.

1 $$f\left({\varvec{P}}\right)=\frac{1}{32}\sum_{x=1}^{32}{\omega }_{c}{\mu }_{c}\left({{\varvec{p}}}_{{\varvec{x}}}\right)+{\omega }_{p}{\mu }_{p}\left({{\varvec{p}}}_{{\varvec{x}}}\right)$$

where, ${\varvec{P}}=\left\{{{\varvec{p}}}_{1},{{\varvec{p}}}_{2},\dots ,{{\varvec{p}}}_{32}\right\}$ is the combination pattern (T1 to T32), *ω*_*c*_ and *ω*_*p*_ are weights of confidence score and coordinate score, respectively. ${\mu }_{c}\left({{\varvec{p}}}_{{\varvec{x}}}\right)$ is confidence score of candidate ${{\varvec{p}}}_{{\varvec{x}}}$ obtained from faster R-CNN in the range of [0–1], whereas ${\mu }_{p}\left({{\varvec{p}}}_{{\varvec{x}}}\right)$ is the positional relationship score of candidate ${{\varvec{p}}}_{{\varvec{x}}}$ calculated by using the following equation:

2 $${\mu }_{p}\left({{\varvec{p}}}_{{\varvec{x}}}\right)=\frac{1}{\left|\Omega \right|}{\sum }_{{{\varvec{p}}}_{{\varvec{y}}}\in{\varvec{\Omega}}}\delta \left({{\varvec{p}}}_{{\varvec{x}}},{{\varvec{p}}}_{{\varvec{y}}}\right)$$

$${\varvec{\Omega}}=\{{{\varvec{p}}}_{{\varvec{x}}+{\varvec{i}}}|i=\left\{-2,-\mathrm{1,1},2\right\},1\le x+i\le 32\}$$

where, ${{\varvec{p}}}_{{\varvec{x}}}$ is the tooth candidate under consideration and $\delta \left({{\varvec{p}}}_{{\varvec{x}}},{{\varvec{p}}}_{{\varvec{y}}}\right)$ is a customized function created using the prior knowledge of the dataset. The function evaluates the horizontal distance between the tooth ${{\varvec{p}}}_{{\varvec{x}}}$ with its neighboring teeth ${{\varvec{p}}}_{{\varvec{y}}}$ [where, ${{\varvec{p}}}_{{\varvec{y}}}={{\varvec{p}}}_{{\varvec{x}}-2},{{\varvec{p}}}_{{\varvec{x}}-1},{{\varvec{p}}}_{{\varvec{x}}+1},{{\varvec{p}}}_{{\varvec{x}}+2}$]. It then calculates and assigns score for each tooth defined as coordinate score following the equation below:

3 $$\delta \left({{\varvec{p}}}_{{\varvec{x}}},{{\varvec{p}}}_{{\varvec{y}}}\right)=\left\{\begin{array}{ll}0& \left|{p}_{x}^{x}-{p}_{y}^{x}\right|<a\\ k+\left(1-k\right)\frac{\left|{p}_{x}^{x}-{p}_{y}^{x}\right|-a}{b-a}& a<\left|{p}_{x}^{x}-{p}_{y}^{x}\right|<b\\ 1& b<\left|{p}_{x}^{x}-{p}_{y}^{x}\right|<c\\ 1-\left(1-k\right)\frac{\left|{p}_{x}^{x}-{p}_{y}^{x}\right|-c}{d-c}& c<\left|{p}_{x}^{x}-{p}_{y}^{x}\right|<d\\ 0& d<\left|{p}_{x}^{x}-{p}_{y}^{x}\right|\\ k& {p}_{x}=\emptyset \, or \, {p}_{y}=\emptyset \end{array}\right.$$

**Figure 3:**
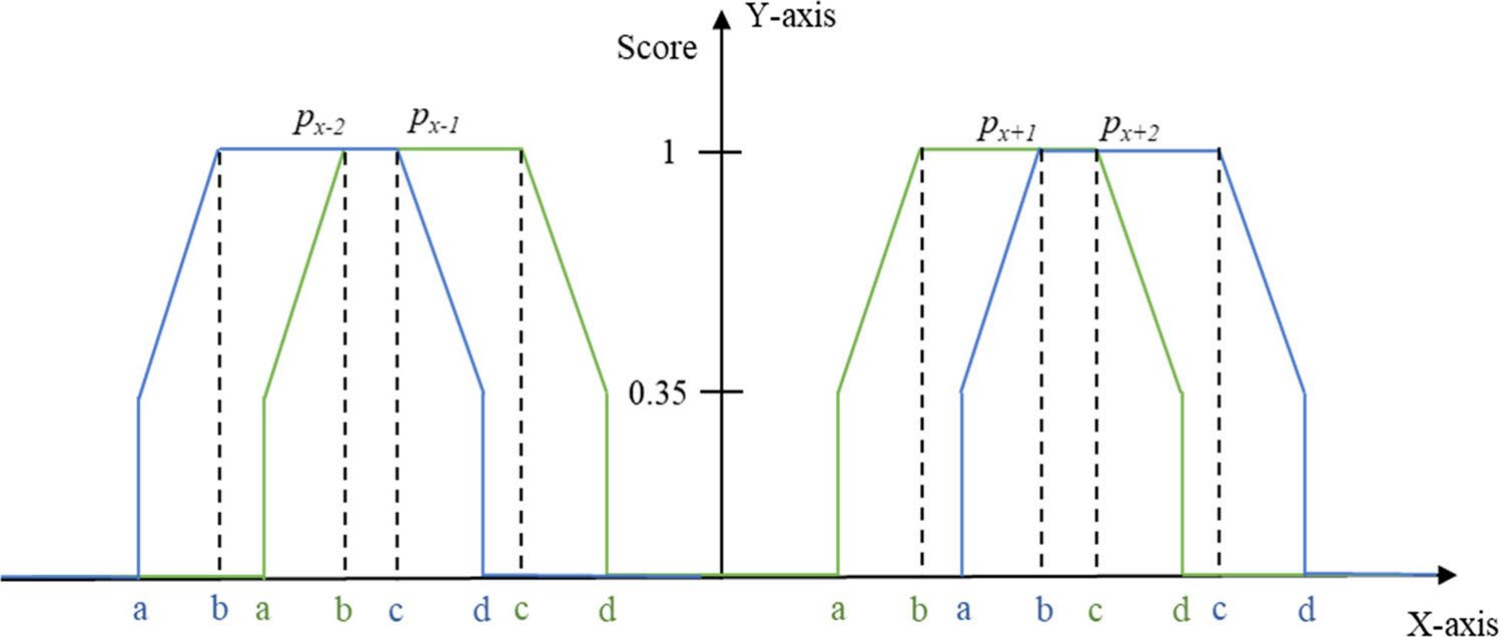
Mechanism of calculating the coordinate score $({{\varvec{\mu}}}_{{\varvec{p}}})$.

${p}_{x}^{x}$ and ${p}_{y}^{x}$ are x-coordinate values of center point of candidate ${{\varvec{p}}}_{{\varvec{x}}}$ and ${{\varvec{p}}}_{{\varvec{y}}}$, respectively. The parameters, *a*, *b*, *c* and *d* determine the shape of the function. The values are determined experimentally and set as (*a*, *b*, *c*, *d*) = (30, 47, 114, 130) when ${{\varvec{p}}}_{{\varvec{y}}}={{\varvec{p}}}_{{\varvec{x}}-1}$ **or** ${{\varvec{p}}}_{{\varvec{x}}+1}$, and (a, b, c, d) = (80, 97, 164, 180) when ${{\varvec{p}}}_{{\varvec{y}}}={{\varvec{p}}}_{{\varvec{x}}-2}$ **or** ${{\varvec{p}}}_{{\varvec{x}}+2}$. The value of *k* is also determined experimentally and set as 0.35 for this experiment. Figure 3 shows the mechanism of calculating the coordinate score. The different colors refer to the different teeth number. The weights of confidence score and coordinate score are two of the parameters of this algorithm and they should be selected carefully. There may have multiple candidates for a single tooth that the optimization algorithm should fix. In that case, the candidates are numbered in accordance with its confidence value, i.e. candidate tooth with higher confidence value will be numbered first. For example, tooth T1 has two candidates with confidence value 0.95 and 0.7, they will be denoted as *C*_*1,1*_ and *C*_*1,2*_, respectively. The first subscript refers to the tooth number. And, ${C}_{x,0}$ represents the missing tooth candidate. The candidate optimization processes in three steps.

*Step 1*: Initialize pattern ${{\varvec{P}}}_{{\varvec{m}}{\varvec{a}}{\varvec{x}}}=\left\{{C}_{\mathrm{1,1}},{C}_{\mathrm{2,1}},\dots ,{C}_{\mathrm{32,1}}\right\}$ by choosing the candidate with the highest confidence value for each tooth. When there are no candidate at tooth *x*, ${C}_{x,0}$ is used as the candidate. The score of pattern ***P***_***max***_ is calculated using Eq. (8), and let the calculated score be *S*_*max*_.

*Step 2*: For every tooth *x*, try all candidates of *C*_*x*_, where *C*_*x*_ = (*C*_*x,0*_, *C*_*x,1*_, *C*_*x, 2*_ …) and calculate the score using Eq. (8). Update *S*_*max*_, if new best combination is found, and set the candidate to ***P***_***tmp.***_

*Step 3*: If there are update for *S*_*max*_, ***P***_***max***_ is replaced by ***P***_***tmp***_ and return to *Step 2*. Else, the algorithm finds the best combination of all.

### Performance analysis

**Figure 4:**
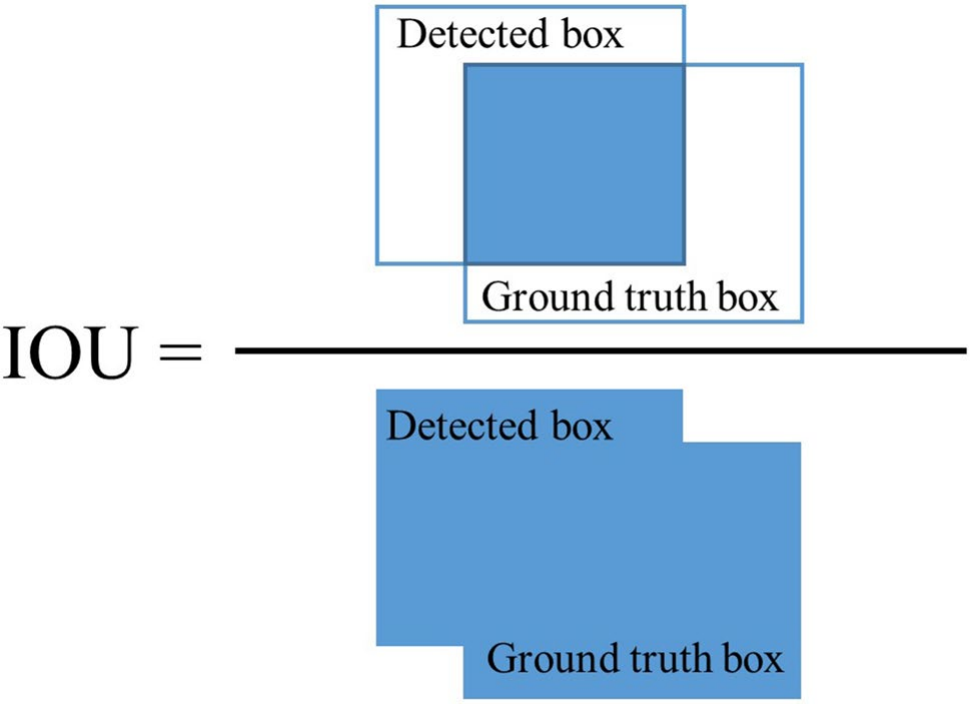
Illustration of intersection-over-union (IOU).

Average precision (AP)^29^ is calculated for each category of tooth to evaluate the candidate detection performance of the proposed method. At first, the detected boxes are compared with the ground truth boxes by calculating the intersection-over-union (IOU) as shown in Fig. 4 and defined as below

4 $$IOU=\frac{Are{a}_{Detecte{d}_{Box}}\cap Are{a}_{GroundTrut{h}_{Box}}}{Are{a}_{Detecte{d}_{Box}}\cup Are{a}_{GroundTrut{h}_{Box}}}$$

The IOU threshold value is set as 0.5 i.e. if the IOU value is greater or equal to 0.5 then the detected box is considered as true positive, otherwise is considered as false positive. To calculate the evaluation index, i.e. AP, precision and recall are calculated using the equations as follows.

5 $$precision= \frac{TP}{TP+FP}$$

6 $$recall= \frac{TP}{TP+FN}$$

where, *TP* is defined as the number of ground truth boxes that overlap with the detected boxes with *IOU* ≥ 0.5; *FP* is defined as the number of detected boxes that overlap with the ground truth boxes with *IOU* < 0.5, and *FN* is defined as the number of teeth that are not detected or detected with *IOU* < 0.5. Finally, the model is tested with a test dataset of 100 images. The above mentioned metrics are used to evaluate the detected boxes.

**Figure 5:**
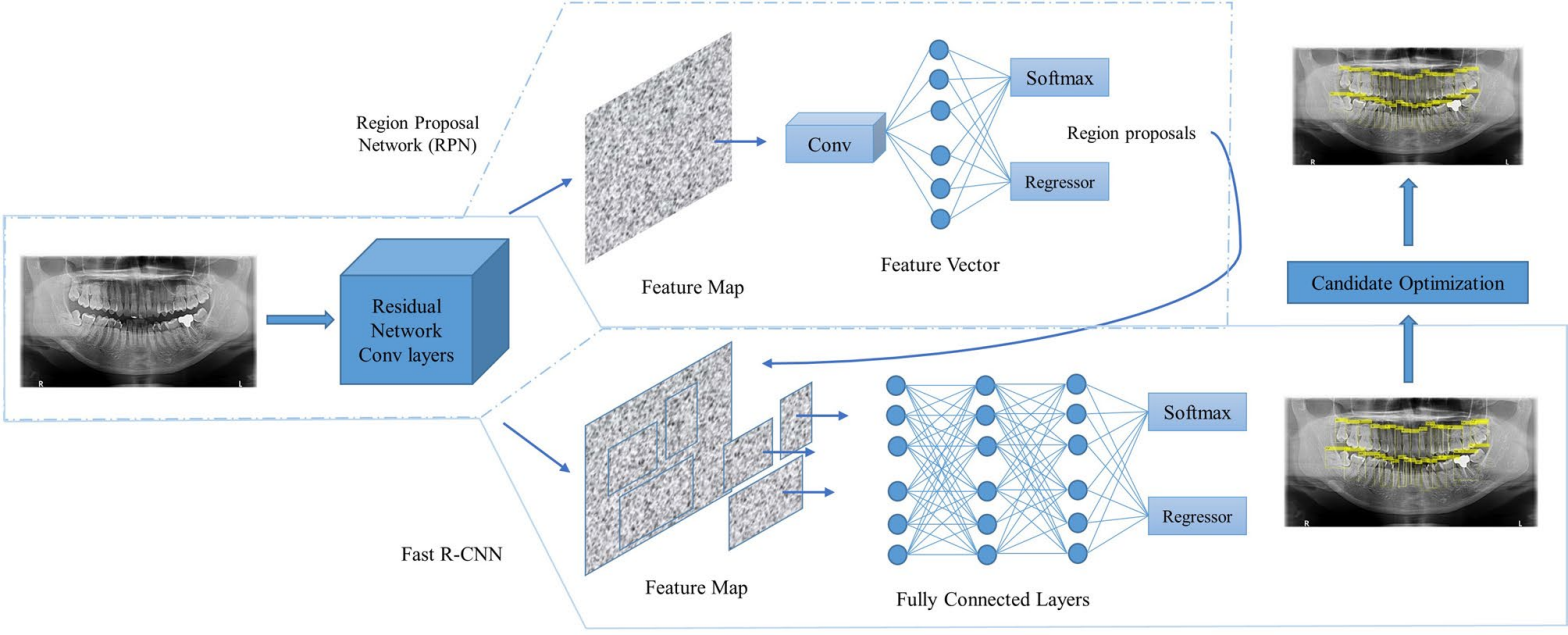
Illustration of proposed teeth recognition model.

The overall proposed model with candidate optimization algorithm is depicted in Fig. 5. To evaluate the performance of candidate optimization algorithm, *F*_*1*_ score is calculated. *F*_*1*_ score is the harmonic mean of precision and recall and is defined by the following equation:

7 $${F}_{1 }score=\frac{2\times (precision\times recall)}{(precision+recall)}$$

## Results and discussion

This section presents the overall simulation results using residual network based faster R-CNN for teeth recognition. The proposed method was implemented in MATLAB 2019a software and executed with Ryzen 7 2700 Eight-Core Processors (16 CPUs) with clock speed ~ 3.2 GHz. The training and testing were done with TITAN RTX 24 GB display memory (VRAM). Table 2 refers to the parameter settings of faster R-CNN for teeth recognition task. Total number of epoch was set as 10. As each training image corresponds to each iteration, thus total number of iteration was 9000. Number of regions to sample from each training image was set as 256, whereas number of strongest regions to be used for generating training samples was set as 2000. Negative overlap range and positive overlap range were set as [0–0.3] and [0.6–1], respectively. To better explain the results of the proposed model, total three cases were considered.

**Table 2:** Parameter settings of faster R-CNN.

Parameter	Value
Epoch	10
Iteration	9000
Initial learning rate	0.001
Mini batch size	1
Number of regions to sample	256
Number of strongest regions	2000
Negative overlap range	[0–0.3]
Positive overlap range	[0.6–1]

**Figure 6:**
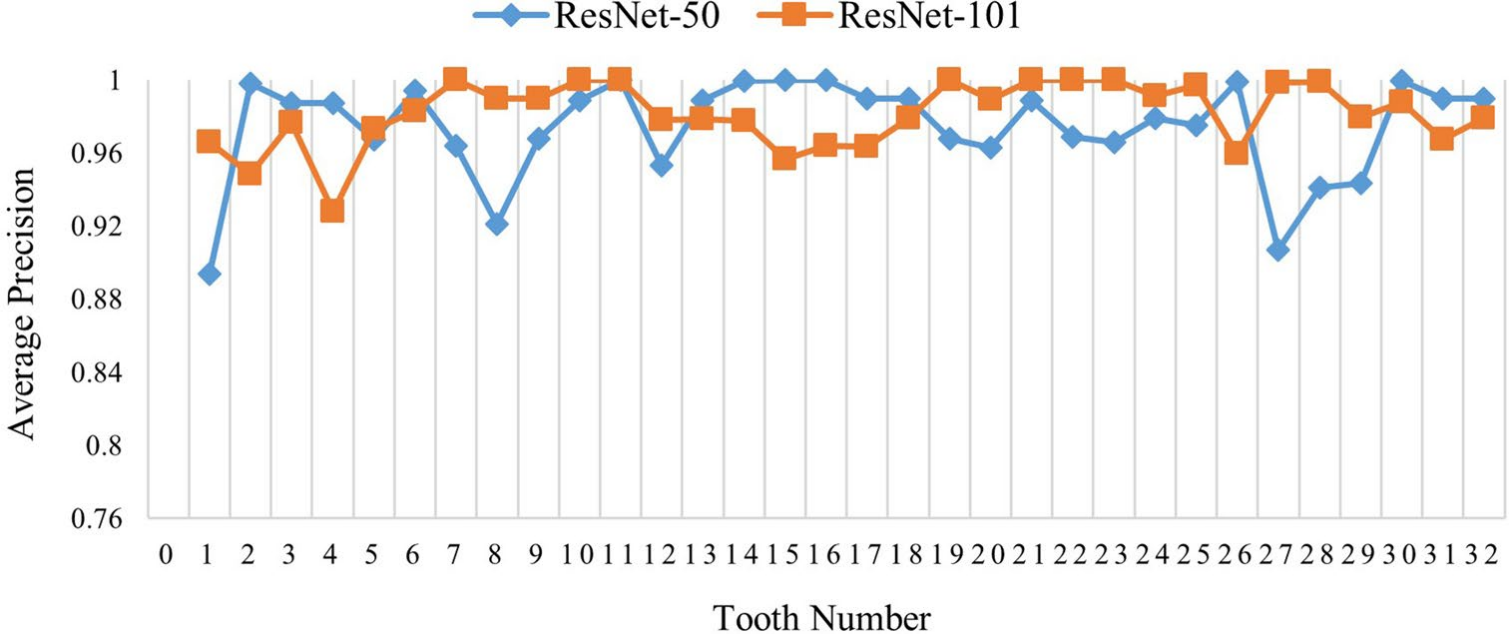
Average precision for different tooth category.

**Figure 7:**
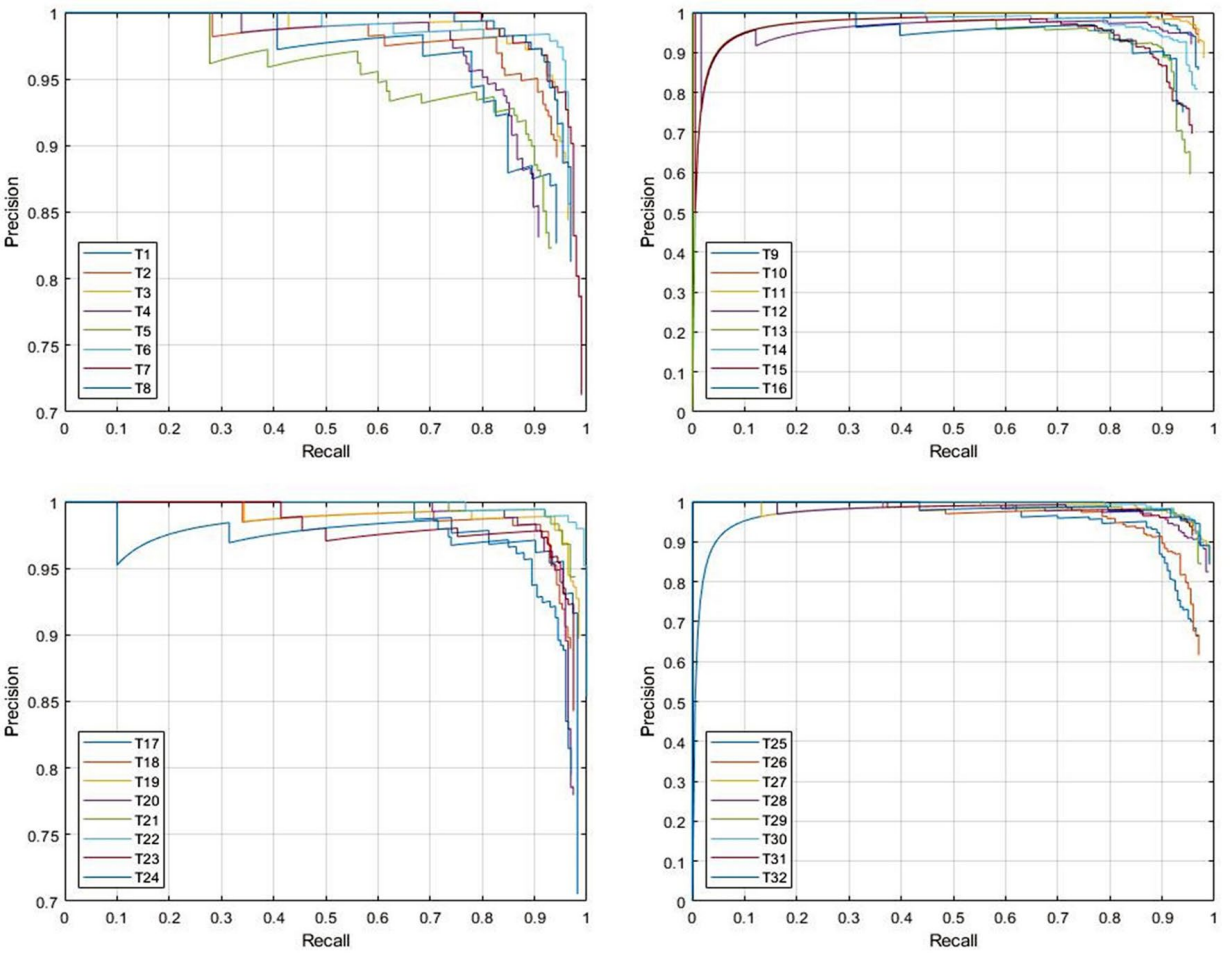
Recall-precision curve of different teeth categories for ResNet-101. The curves were generated by MATLAB 2019a software.

*Test case 1*: Total 900 panoramic radiographs were used to train the network, while 100 images were used for testing. Both, ResNet-50 and ResNet-101 networks were implemented separately as the base networks of faster R-CNN for the evaluation purpose. Figure 6 is presenting the comparison of candidate detection results between ResNet-50 and ResNet-101 for each tooth category. It can be seen from the Fig. 7 that only T1 achieves less than 0.900 AP while using ResNet-50. Other than that the AP of other teeth is above 0.900 and the mAP is 0.974. On the other hand, results obtained using ResNet-101 shows extremely good detection performance as total seven teeth categories achieve maximum average precision and mAP is 0.981, which is better than the results obtained by ResNet-50. Table 3 presents precision and recall value for each tooth category. Some of the teeth categories achieved perfect recall values for ResNet-101 based faster R-CNN i.e. there were no false negative for those teeth categories. Figure 7 shows recall-precision curves for different teeth categories using ResNet-101. For better visualization, the curves are shown in four different figures, and each figure shows recall-precision curves for eight categories of teeth. Almost all of the curves show ideal behavior and visibly it can be seen that the convergence performance is close to that of an ideal one. The recognition performance obtained by the proposed model is close to the level of an expert dentist.

**Table 3:** Recall and precision value for each tooth category.

Tooth number	ResNet-50		ResNet-101		Tooth number	ResNet-50		ResNet-101	
	Precision	Recall	Precision	Recall		Precision	Recall	Precision	Recall
T1	0.8876	0.9186	0.9231	0.9796	T17	0.9474	0.9643	0.9565	0.9706
T2	0.8585	0.9529	0.9400	0.9592	T18	0.9381	0.9529	0.9798	0.9798
T3	0.9272	0.9745	0.9412	0.9796	T19	0.9458	0.9846	1.0000	1.0000
T4	0.9785	0.9333	0.9479	0.9286	T20	0.9234	0.9847	0.9899	0.9899
T5	0.8788	0.9667	0.8788	0.9775	T21	0.9641	0.9947	0.9495	1.0000
T6	0.9598	0.9695	0.9700	0.9898	T22	0.9802	1.0000	1.0000	1.0000
T7	0.9469	0.9899	1.0000	1.0000	T23	0.9369	0.9747	0.9900	1.0000
T8	0.9327	0.9848	0.9706	0.9900	T24	0.8977	0.9650	0.9174	1.0000
T9	0.9557	0.9848	0.9703	0.9899	T25	0.8122	0.9300	0.9901	1.0000
T10	0.9423	0.9849	0.9802	1.0000	T26	0.9234	0.9650	0.9600	0.9600
T11	0.9897	0.9747	0.9802	1.0000	T27	0.9557	0.9848	0.9604	1.0000
T12	0.9267	0.9779	0.9375	0.9783	T28	0.9444	0.9791	0.9505	1.0000
T13	0.9439	0.9536	0.9604	0.9798	T29	0.9200	0.9583	0.9798	0.9798
T14	0.8720	0.9787	0.9314	0.9794	T30	0.9444	0.9791	0.9697	0.9897
T15	0.8488	0.9305	0.8727	0.9697	T31	0.9109	0.9684	0.9691	0.9691
T16	0.9036	0.9036	0.8393	0.9792	T32	0.9211	0.9722	0.9688	0.9841

**Figure 8:**
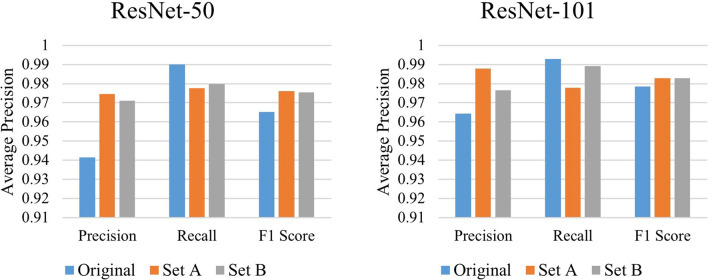
Comparison of results before and after applying candidate optimization algorithm (Set A: *ω*_*c*_ = 0.8, *ω*_*p*_ = 0.2; Set B: *ω*_*c*_ = 0.5, *ω*_*p*_ = 0.5).

*Test case 2*: Second test case examines the compatibility and the feasibility of using candidate optimization algorithm along with faster R-CNN technique to improve overall recognition result. The candidate optimization algorithm based on prior knowledge was implemented along with faster R-CNN technique to refine the obtained detected boxes by faster R-CNN. In this paper, two sets of weights were considered. In set A, weight of confidence score (*ω*_*c*_) was set as 0.8, whereas the weight of positional relationship score (*ω*_*p*_) was set as 0.2. In set B, both weights were selected as 0.5. Table 4 shows the recognition results after applying the candidate optimization algorithm. From the table, it is clear that for both the networks, the overall *F*_*1*_ scores improves. For ResNet-50 and ResNet-101, *F*_*1*_ score improves from 0.965 to maximum 0.976 and 0.978 to 0.983, respectively. The optimization technique also effectively balances the difference between precision and recall, which indicates that the algorithm is fully compatible with the model and successfully improves its robustness. Figure 8 visualizes the result given in Table 3. In terms of *F*_*1*_ score, set A performed better than set B for ResNet-50, whereas it remained in balance for ResNet-101.

**Table 4:** Recognition results after applying candidate optimization algorithm.

	ResNet-50			ResNet-101		
	Original	*ω*_*c*_ = 0.8 *ω*_*p*_ = 0.2	*ω*_*c*_ = 0.5 *ω*_*p*_ = 0.5	Original	*ω*_*c*_ = 0.8 *ω*_*p*_ = 0.2	*ω*_*c*_ = 0.5 *ω*_*p*_ = 0.5
Precision	0.942	**0.975**	0.971	0.964	**0.988**	0.977
Recall	**0.990**	0.978	0.980	**0.993**	0.978	0.989
*F*_*1*_ Score	0.965	**0.976**	0.975	0.978	**0.983**	**0.983**

Bold values indicate the best results in that particular row (particular section).

**Figure 9:**
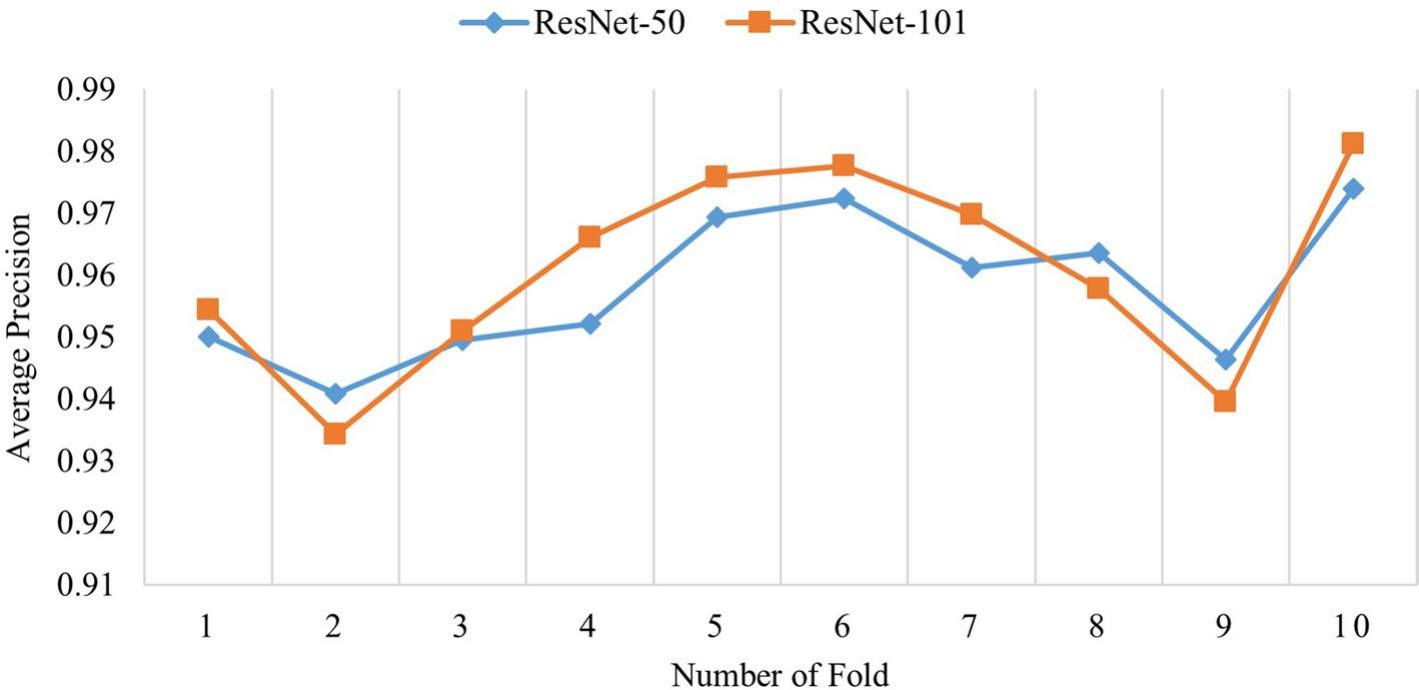
Mean average precision (mAP) for different folds in tenfold cross validation.

*Test case 3*: In order to check the robustness of the proposed method, K-fold cross validation (CV) technique was utilized in this experiment. In this paper, K is equal to 10 i.e. the whole dataset is divided into 10 different folds. Total 10 runs were required to perform tenfold CV. Both, ResNet-50 and ResNet-101 were used with faster R-CNN, separately to perform the recognition task. The overall results consisting of AP of all teeth categories in all 10 test datasets for ResNet-50 and ResNet-101 are summarized in Fig. 9. The obtained results of 10-CV presented in Fig. 9 shows that the residual network based faster R-CNN performed quite strongly and consistently with the average of mAP is 0.958 for ResNet-50 and 0.960 for ResNet-101. Furthermore, the robustness of the method in different test data shows that it is clinically applicable. The comparison of different residual networks presented in Fig. 9 shows ResNet-101 performs better for 7 folds, whereas ResNet-50 performs better in 3 other folds. The lowest AP achieved by ResNet-50 is 0.800 for T27 in K7 fold and 0.840 for T1 in K7 fold by ResNet-101. However, most of the lower detections came from the eighth number fold (i.e. K8) for both of the residual networks. To assess the feasibility and robustness of the proposed model after applying candidate optimization algorithm, tenfold CV technique was performed and the results are presented in Table 5. After applying candidate optimization, the average *F*_*1*_ score improves from 0.962 to 0.971 for ResNet-50 and 0.975 to 0.976 for ResNet-101, respectively. Furthermore, for all cases, the candidate optimization algorithm refined the detected boxes successfully and thus, improved the overall recognition performance.

**Table 5:** Results (in *F*_*1*_ score) of teeth recognition after applying candidate optimization algorithm for tenfold cross validation.

	Number of teeth in test dataset	ResNet-50			ResNet-101		
		Original	*ω*_*c*_ = 0.8 *ω*_*p*_ = 0.2	*ω*_*c*_ = 0.5 *ω*_*p*_ = 0.5	Original	*ω*_*c*_ = 0.8 *ω*_*p*_ = 0.2	*ω*_*c*_ = 0.5 *ω*_*p*_ = 0.5
K1	2947	0.957	0.969	**0.971**	0.975	0.976	**0.977**
K2	2890	0.962	0.967	**0.967**	0.967	0.969	**0.970**
K3	2904	0.961	0.969	**0.969**	0.970	0.973	**0.974**
K4	2909	0.962	**0.969**	**0.969**	0.967	**0.972**	0.972
K5	2958	0.974	**0.980**	0.979	0.982	**0.984**	0.984
K6	2847	0.958	**0.969**	0.969	0.971	**0.975**	0.974
K7	2862	0.974	0.983	**0.984**	0.984	0.987	**0.987**
K8	2796	0.956	0.961	**0.963**	0.964	**0.967**	0.967
K9	2846	0.951	0.962	**0.964**	0.962	0.968	**0.970**
K10	2969	0.965	**0.976**	0.975	0.978	**0.983**	**0.983**
**Average**	2893	0.962	0.971	**0.971**	0.972	0.975	**0.976**

Bold values indicate the best results in that particular row (particular section).

**Figure 10:**
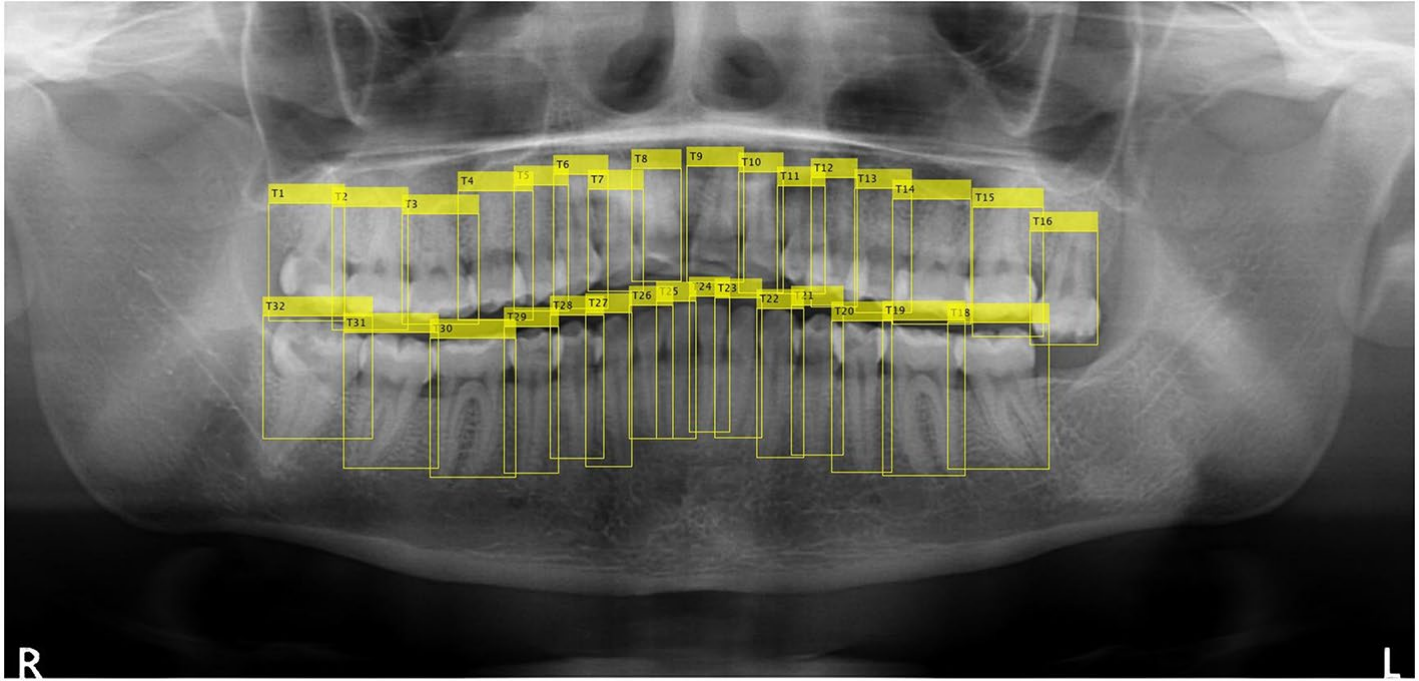
Successful teeth detection in noisy panoramic radiograph. The detected boxes were generated by MATLAB 2019a software.

The stand-alone residual network based faster R-CNN performed exceedingly well in recognizing tooth by its number. Two kinds of residual networks i.e. ResNet-50 and ResNet-101 were used as base networks of faster R-CNN. ResNet-101 is deeper network than ResNet-50 and it performs marginally better than its shallower counterpart. Although, ResNet-101 performs better in terms of recognition, it is computationally costlier than ResNet-50. As their recognition performance is not much different, authors recommend using ResNet-50 as a base network when computational cost is a concern. The inclusion of candidate optimization algorithm further improves the recognition performance of the proposed model. However, the optimization parameters should be chosen carefully based on the dataset in order to have a good impact on the overall recognition results. Figure 10 shows detected teeth in a noisy panoramic radiograph. This research excludes severely broken teeth from the experiment.

## Conclusion

This research proposes a method for automatic teeth recognition in dental panoramic radiographs. The method is based on candidate detection with residual network based faster R-CNN and candidate optimization using a prior knowledge. Two versions of residual network i.e. ResNet-50 and ResNet-101 are used as base networks for faster R-CNN separately. The combination of residual network with faster R-CNN method successfully performs the recognition task with a high degree of accuracy. It achieves maximum 0.980 mAP. A prior knowledge based candidate optimization technique is also incorporated to improve the overall recognition performance. The introduction of the optimization method improves the *F*_*1*_ score from 0.965 to maximum 0.976 and 0.978 to 0.983 for ResNet-50 and ResNet-101, respectively. The K-fold cross validation technique is also implemented with and without candidate optimization technique that effectively verifies the feasibility and the robustness of the proposed method. The level of performance achieved by the proposed model is close to an expert dentist and thus, clinically implementable. Finally, it can be said that the proposed model can be used as a reliable and useful tool to assist dental care professionals in dentistry. In future, we plan to extend the current model to include automatic dental condition evaluation and prosthetic detection features.
